# *Neospora caninum* Suspects as One of the Most Important Causes of Abortion in Large Dairy Farms in Isfahan, Iran

**Published:** 2017

**Authors:** Morteza HOSSEININEJAD, Mohammadreza MAHZOUNIEH, Naser SHAMS ESFANDABADI

**Affiliations:** 1. Dept. of Clinical Sciences, Faculty of Veterinary Medicine, Shahrekord University, Shahrekord, Iran; 2. Dept. of Pathobiology, Faculty of Veterinary Medicine, Shahrekord University, Shahrekord, Iran

**Keywords:** *Neospora caninum*, Dairy farms, Cattle, Dogs, Iran

## Abstract

**Background:**

This study aimed to reveal the serological prevalence of *Neospora caninum* in large dairy farms in Isfahan Province, central Iran.

**Methods:**

Serum samples were collected from 1500 cattle living in four large dairy farms in Isfahan Province, Iran during 2014–2015 and examined for anti *N. caninum* IgG antibodies. Overall, 113 serum samples were also collected from the dogs living in these areas; suspecting to be risk factors for this infection. All the serum samples were investigated to find IgG antibodies by using ELISA. Dogs’ sera were also analyzed by indirect fluorescent antibody test.

**Results:**

Totally, 395 out of 1500 bovine samples (26.33%) were positive for *N. caninum*: 34%, 21.61%, 23.03% and 29.01% in four investigated clusters (farms). Infection rate was significantly more in cows with the history of abortion. The infection rate in dogs was 17.69%: (20 out of 113).

**Conclusion:**

The results show a high seroprevalence of the infection and possibly the role of the dogs in horizontal transmission of the infection.

## Introduction

*Neospora caninum* is an apicomplexan protozoan parasite recognized as the most important cause of abortion in cattle in many countries. *N. caninum* causes abortion in both dairy and beef cattle. *N. canium* associated abortions may occur in any age; from 3 months of gestation to the end; mostly at 5–6 months of fetus age. Fetuses may die in utero, be resorbed, mummified, autolyzed, stillborn, born alive with clinical signs, or born clinically normal but persistently infected. Neosporosis-induced abortions occur year-round (1).

Dairy and beef cattle with antibodies to *N. caninum* (seropositive) are more likely to abort than seronegative cows (2–4) and up to 95% of calves from seropositive mothers will be congenitally infected without any detectable clinical signs (5).

Dogs (*Canis lupus familiaris*), coyotes (*C. latrans*) and dingos (*C. lupus dingo*) are definitive and intermediate hosts and shed oocysts following ingesting *N. caninum* infected tissues of intermediate hosts (6–8). The presence either of dogs, currently or even within past 10 yr is a risk factor for detecting seropositive cattle (9).

This study was aimed to reveal the serological prevalence of *N. caninum* in large dairy farms in Isfahan Province, Iran, to realize if the parasite is an important agent to induce abortion in these herds and finally if infected dogs are important in seroepidemiology of the infection.

## Materials and Methods

### Animals

A total of 1500 dairy cows were enrolled in this study. All of them were at least 6 months old. The samples were taken from 4 large dairy farms located in Isfahan Province, Iran. Blood samples were taken and centrifuged immediately.

Serum samples were also collected from 113 dogs in the region in which dairy farms were located. Collected sera were kept in −20 °C until used.

### ELISA

An *N. caninum* 38 kDa surface antigen (P38) was affinity purified as described earlier (10) and lyophilized until used for coating the ELISA plates.

To analyses the serum samples taken from dairy cows, the affinity-purified antigen was diluted in coating buffer (0.1 M sodium bicarbonate, pH 8.3) and used to coat ELISA plates (Nunc-Immuno (Polysorb)) at 37 °C for 1 hour. Wells were then washed three times with PBS-T (PBS, pH 7.2, 0.05% Tween-20) and incubated with blocking solution (PBS-T, 20% horse serum) at 37 °C for 0.5 hours. Wells were emptied and the serum samples (diluted 1:200 in PBST, 20% horse serum) were added. Positive and negative controls were kindly provided by Friedrich Loeffler Institute, Wusterhausen Germany. Positive control was taken from an experimentally infected heifer (heifer 44) and negative control was taken from cow 24, before infection (11). Sera were emptied and ELISA plates were washed. Anti-bovine IgG conjugates were diluted in PBST-2% horse serum and incubated (37 °C, 30 min). After each step, the wells were washed three times with PBS-T.

After the final step, the plates were washed three times with PBS-T and twice with distilled water. Bound antibodies were detected by incubation with a substrate containing 100 mg/ml 3,3′,5,5′-tetramethylbenzidine and 0.004% hydrogen peroxide in 0.2 M sodium acetate and 0.2 M citric acid at 37 °C. The reaction was stopped after 15 min by adding sulfuric acid to a final concentration of 2 N, and optical density (OD) values were measured at 492 nm on an ELISA reader.

Sample index values were calculated by the formula SIn= (Sn-N)/(P-N) where SIn is the individual ELISA index value, Sn is the OD value obtained for a single sample, N is the OD value obtained for the negative serum, and P represents the OD values obtained for the positive serum. Sin values of more than 0.153 were regarded positive (12). Evaluation of the serum samples taken from dogs was performed as described earlier (10).

### IFAT

Indirect fluorescent antibody test was performed as described previously for serum samples collected from the dogs. IFAT results were regarding positive when the reaction result was positive at least in dilution of 1:16 (10).

## Results

Serological evaluation of sera taken from four dairy farms revealed that all the farms had seropositive cows against *N. caninum* with the mean infection rate of 26.33% (395 out of 1500 samples). The infection rates of four dairy farms were 34% (84 out of 247), 21.61% (67 out of 310), 23.03% (114 out of 495) and 29.01% (130 out of 448).

**Fig. 1:**
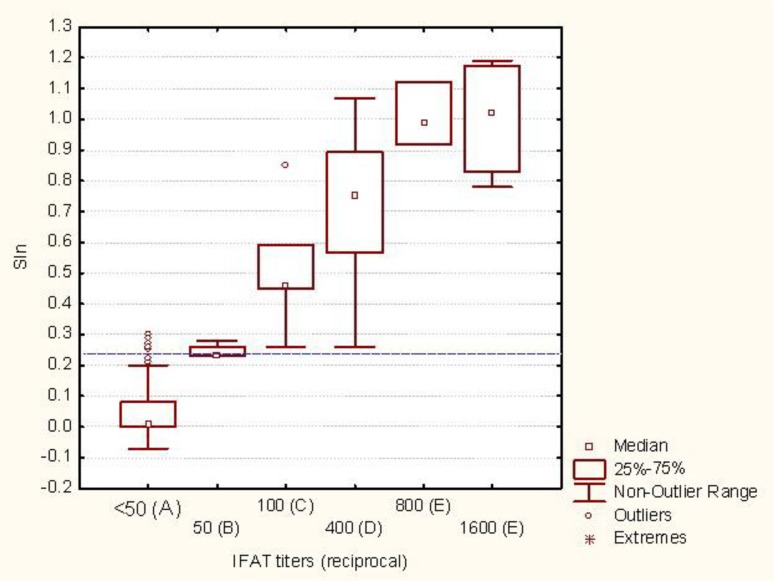
ELISA indices of dogs’ sera in comparison to the IFAT titers

Serological rate of the infection was compared in cows with the history of abortion and cows with no detected abortion. The rate of *N. caninum* infection was significantly higher in cows with the history of abortion (*P*<0.05). Evaluation of dog sera showed that 20 of 113 (17.69%) dogs had detectable anti-*N. caninum* antibodies. The IFAT titers of dog’s sera were compared to the ELISA indices (Fig. 1).

## Discussion

Seroepidemiology of *N. caninum* has been investigated worldwide in dogs, dairy cattle, beef cattle, other domestic animals, wildlife and zoo animals and human (9). Although each serological method has its properties and the results, of one’s serological investigation cannot be compared with the others but overall the results show that many species of mammals are exposed to this parasite worldwide. Minor cross reactivity may occur between *N. caninum* and *Toxoplasma gondii* although it can be ignored (9).

The prevalence of 26.33% observed in this study is relatively high. In another study in Iran, *N. caninum* seroprevalence showed a considerable difference. The prevalence of infection was investigated in Khorasan was 18% and 21% from Ahwaz although it was 44% in another study performed in Khorasan, Iran (13–15). Prevalence of infection is more than what has been reported from neighbor countries (16, 17) and from most of European countries although there are huge differences reported from different regions of one country (9). There are significant differences among different countries, cities, regions, and between beef and dairy cattle. However, differences in serologic techniques, study design, and sample size used should be kept in mind when evaluating these results. Results show that different regions, within a particular region, and among different management systems infection risk differs.

This study showed that the rate of abortion was more in cows with the history of abortion. This is nearly a common finding in other serological studies (2, 3).

There are two patterns of *N. caninum* associated abortions. In the first pattern named *epidemic pattern*, the cause of abortion is postnatal infection of un-infected cattle; most likely due to exposure to the food or water sources contaminated with oocysts (18, 19). This pattern of abortion may lead to abortion storms reported 57% of pregnant cows abort just in few weeks-months (1, 20, 21). If the abortion rate exceeds 10%, 12.5% or 15% of cows at risk within 4, 6 or 8 wk, the abortion will be epidemic (3, 19, 22). Dog feces can represent an important source for parasite-infection. From the time that the dogs were recognized as the definitive hosts for *N. caninum*, the question of its importance in the epidemiology of related abortion was raised (23). The introduction of a new dog in a cattle herd has been reported to increase the risk of epidemic *N. caninum*-related abortion. The newly coming dog was infected with *N. caninum* by infected materials from cattle. It transmitted the infection to other cattle by oocysts shedding (23).

The second pattern of *N. caninum* associated abortion is endemic abortion in which the major route of infection is vertical (19). The re-activation of latent infection during gestation is the reason for an increased abortion risk (24). Chronically infected seropositive cattle have an about two to three fold more risk of abortion compared to seronegative ones (25). Less than 5 percent of cows may have repeated abortion due to neosporosis (26). *N. caninum* transmits vertically in cattle for several generations (27). Most abortions are endemic and most likely due to vertical transmission (1).

## Conclusion

Relatively high *N. caninum* seroprevalence in dogs living inside or in the region where the farms are located suggests the role of dogs to transmit the parasite horizontally. A wide study is needed to detect the exact role of the dogs in the epidemiology of the infection in these farms.

## References

[1] Dubey JP Schares G Diagnosis of bovine neosporosis. Vet Parasitol. 2006; 140(1–2):1–34. 16730126 10.1016/j.vetpar.2006.03.035

[2] Davison HC Otter A Trees AJ Significance of *Neospora caninum* in British dairy cattle determined by estimation of seroprevalence in normally calving cattle and aborting cattle. Int J Parasitol. 1999; 29(8):1189–94. 10576570 10.1016/s0020-7519(99)00094-6

[3] Moen AR Wouda W Mul MF Increased risk of abortion following *Neospora caninum* abortion outbreaks: a retrospective and prospective cohort study in four dairy herds. Theriogenology. 1998; 49(7):1301–9. 10732067 10.1016/S0093-691X(98)00077-6

[4] Thurmond MC Hietala SK Effect of congenitally acquired *Neospora caninum* infection on risk of abortion and subsequent abortions in dairy cattle. Am J Vet Res. 1997; 58(12):1381–5. 9401685

[5] Paré J Thurmond MC Hietala SK Congenital *Neospora caninum* infection in dairy cattle and associated calfhood mortality. Can J Vet Res. 1996; 60(2):133–9. 8785719 PMC1263819

[6] McAllister MM Dubey JP Lindsay DS Dogs are definitive hosts of *Neospora caninum*. Int J Parasitol. 1998; 28 (9):1473–8. 9770635

[7] Gondim LF McAllister MM Pitt WC Coyotes (*Canis latrans*) are definitive hosts of *Neospora caninum*. Int J Parasitol. 2004; 34(2):159–61. 15037103 10.1016/j.ijpara.2004.01.001

[8] King JS Slapeta J Jenkins DJ Australian dingoes are definitive hosts of *Neospora caninum*. Int J Parasitol. 2010; 40(8):945–50. 20149793 10.1016/j.ijpara.2010.01.008

[9] Dubey JP Schares G Ortega-Mora LM Epidemiology and control of neosporosis and *Neospora caninum*. Clin Microbiol Rev. 2007; 20 (2):323–67. 17428888 10.1128/CMR.00031-06 PMC1865591

[10] Hosseininejad M Hosseini F Mosharraf M Development of an indirect ELISA test using an affinity purified surface antigen (P38) for sero-diagnosis of canine *Neospora caninum* infection. Vet Parasitol. 2010; 171(3–4):337–42. 20434268 10.1016/j.vetpar.2010.04.003

[11] Schares G Rauser M Zimmer K Serological differences in *Neospora caninum*-associated epidemic and endemic abortions. J Parasitol. 1999; 85(4):688–94. 10461950

[12] Schares G Rauser M Söndgen P Use of purified tachyzoite surface antigen p38 in an ELISA to diagnose bovine neosporosis. Int J Parasitol. 2000; 30(10):1123–30. 10996331 10.1016/s0020-7519(00)00092-8

[13] Razmi GR Mohammadi GR Garrosi T Seroepidemiology of *Neospora caninum* infection in dairy cattle herds in Mashhad area, Iran. Vet Parasitol. 2006;135(2):187–9. 16289861 10.1016/j.vetpar.2005.09.004

[14] Sadrebazzaz A Haddadzadeh H Esmailnia K Serological prevalence of *Neospora caninum* in healthy and aborted dairy cattle in Mashhad, Iran. Vet Parasitol. 2004; 124(3–4):201–4. 15381300 10.1016/j.vetpar.2004.06.027

[15] Hajikolaei MR Hamidinejat H Ghorbanpoor M Serological study of *Neospora caninum* infection in cattle from Ahvaz area, Iran. Int J Vet Res. 2008; 2 (1):63–6, 127.

[16] Sevgili M Atla MG Keskin O Seroprevalence of *Neospora caninum* in cattle in the province of anlurfa. Turk J Vet Anim Sci. 2005; 29 127–30.

[17] Akca A Gokce HI Guy CS Prevalence of antibodies to *Neospora caninum* in local and imported cattle breeds in the Kars province of Turkey. Res Vet Sci. 2005; 78 (2):123–6. 15563918 10.1016/j.rvsc.2004.08.006

[18] McAllister MM Björkman C Anderson-Sprecher R Evidence of point-source exposure to *Neospora caninum* and protective immunity in a herd of beef cows. J Am Vet Med Assoc. 2000; 217(6):881–7. 10997162 10.2460/javma.2000.217.881

[19] Schares G Bärwald A Staubach C P38-avidity-ELISA: examination of herds experiencing epidemic or endemic *Neospora caninum*-associated bovine abortion. Vet Parasitol. 2002; 106(4):293–305. 12079735 10.1016/s0304-4017(02)00103-6

[20] Jenkins MC Caver JA Björkman C Serological investigation of an outbreak of *Neospora caninum*-associated abortion in a dairy herd in southeastern United States. Vet Parasitol. 2000; 94(1–2):17–26. 11078940 10.1016/s0304-4017(00)00373-3

[21] McAllister MM Huffman EM Hietala SK Evidence suggesting a point source exposure in an outbreak of bovine abortion due to neosporosis. J Vet Diagn Invest. 1996; 8 (3):355–7. 8844580 10.1177/104063879600800313

[22] Wouda W Bartels CJ Moen AR Characteristics of *Neospora caninum*-associated abortion storms in dairy herds in The Netherlands (1995 to 1997). Theriogenology. 1999; 52(2):233–45. 10734391 10.1016/s0093-691x(99)00125-9

[23] Dijkstra T Barkema HW Eysker M Natural transmission routes of *Neospora caninum* between farm dogs and cattle. Vet Parasitol. 2002; 105 (2):99–104. 11900923 10.1016/s0304-4017(02)00010-9

[24] Guy CS Williams DJL Kelly DF *Neospora caninum* in persistently infected, pregnant cows: spontaneous transplacental infection is associated with an acute increase in maternal antibody. Vet Rec. 2001; 149 (15):443–9. 11688746 10.1136/vr.149.15.443

[25] Wouda W Moen AR Schukken YH Abortion risk in progeny of cows after a *Neospora caninum* epidemic. Theriogenology. 1998; 49(7):1311–6. 10732068 10.1016/S0093-691X(98)00078-8

[26] Anderson ML Palmer CW Thurmond MC Evaluation of abortions in cattle attributable to neosporosis in selected dairy herds in California. J Am Vet Med Assoc. 1995; 207(9):1206–10. 7559072

[27] Schares G Peters M Wurm R The efficiency of vertical transmission of *Neospora caninum* in dairy cattle analyzed by serological techniques. Vet Parasitol. 1998; 80(2):87–98. 9870361 10.1016/s0304-4017(98)00195-2

